# TRPM7 via calcineurin/NFAT pathway mediates metastasis and chemotherapeutic resistance in head and neck squamous cell carcinoma

**DOI:** 10.18632/aging.204154

**Published:** 2022-06-29

**Authors:** Tsung-Ming Chen, Chih-Ming Huang, Ming-Shou Hsieh, Chun-Shu Lin, Wei-Hwa Lee, Chi-Tai Yeh, Shao-Cheng Liu

**Affiliations:** 1 Department of Otolaryngology-Head and Neck Surgery, Shuang Ho Hospital, Taipei Medical University, New Taipei City 23561, Taiwan; 2 Department of Otolaryngology, School of Medicine, College of Medicine, Taipei Medical University, Taipei City 11031, Taiwan; 3 Department of Otolaryngology, Taitung Mackay Memorial Hospital, Taitung City 950408, Taiwan; 4 Department of Nursing, Tajen University, Yanpu 90741, Pingtung County, Taiwan; 5 Department of Medical Research and Education, Taipei Medical University - Shuang Ho Hospital, New Taipei City 235, Taiwan; 6 Department of Radiation Oncology, Tri-Service General Hospital, National Defense Medical Center, Taipei City 114, Taiwan; 7 Department of Pathology, Taipei Medical University-Shuang Ho Hospital, New Taipei City 235, Taiwan; 8 Department of Medical Laboratory Science and Biotechnology, Yuanpei University of Medical Technology, Hsinchu City 30015, Taiwan; 9 Department of Otolaryngology-Head and Neck Surgery, Tri-Service General Hospital, National Defense Medical Center, Taipei City 114, Taiwan

**Keywords:** HNSCC, transient receptor potential cation channel subfamily M member 7, chemoresistance, calcineurin/NFAT pathway, cancer stem cell

## Abstract

The exact mechanisms of Head and neck squamous carcinoma (HNSCC) chemoresistance and metastatic transformation remain unclear. In recent decades, members of the transient receptor potential (TRP) channel family have been proposed as potential biomarkers and/or drug targets in cancer treatment. First, in a TCGA cohort of HNSCC, TRPM7 is highly expressed in cancer tissues, especially the expression in invasive cancer tissues is statistically significant (p>0.001). In GEO and TCGA cohort, patients with high expression of TRPM7 and NFATC2 have poor overall survival rates. The expression of TRPM7 and NFATC2 showed a positive correlation. Compared to human normal oral keratinocytes (hNOK), TRPM7 is overexpressed in FaDU, SAS, and TW2.6 cell lines. Similarly, patients with HNSCC exhibited higher TRPM7 expression than non-HNSCC subjects, and this high TRPM7 expression was associated with worse 5-year overall survival. Furthermore, TRPM7 inversely correlated with E-cadherin, but positively correlated with Vimentin, NANOG, and BMI-1 mRNA levels. Consistent with this, we demonstrated the overexpression of TRPM7 in cisplatin-resistant subjects, compared to the cisplatin-sensitive counterparts. Moreover, shRNA-mediated silencing of TRPM7 significantly suppressed the migration, invasion, colony formation, and tumorsphere formation of SAS cells, with associated downregulation of Snail, c-Myc, cyclin D1, SOX2, OCT4, and NANOG proteins expression. Finally, compared with the untreated wild-type SAS cells or cisplatin-treated cells, shTRPM7 alone or in combination with cisplatin significantly inhibited tumorsphere and colony formation. These findings serving as the basis for development of novel therapeutic strategies against metastasis and chemoresistance, while providing new insights into TRPM7 biology and activity in HNSCC.

## INTRODUCTION

Despite improvements in radiotherapy and surgical techniques for managing advanced head and neck squamous cell carcinoma (HNSCC) over the past two decades as well as with the enhanced treatment efficacy of chemotherapy and monoclonal antibodies, more than half of all treated HNSCC patients experience disease recurrence [1–4]. In addition to disease recurrence, metastasis to the cervical lymph node is a critical prognostic factor for HNSCC, with uncontrolled metastasis and local recurrence being increasingly implicated in the low overall survival rates in the last two decades despite diagnostic and therapeutic advancements [5]. Oncogene overexpression profiles in the original primary tumor site may facilitate and thus predict the incidence of lymph node metastasis, making them potential biomarkers of HNSCC progression [4]. Identifying these genetic or molecular factors involved in disease progression may improve our concordat of HNSCC malignant and is essential for developing treatment strategies to improve patient survival.

Ion channels are often overexpressed in tumor cells, stromal cells, and endothelial cells. Studies have shown that ion channels are involved in cancer-related processes, including tumor angiogenesis and metastasis. For instance, tumor formation involves changes in pH, which are associated with proton exchange and water transport across the plasma membrane. Neovascularization, associated with Ca^2+^, K^+^, and Na^+^ channels, is a necessary component of subsequent tumor growth and distant metastasis. Furthermore, ion channels are key mechanisms that control the cellular volume and maintain the membrane potential [6]. Recent evidence indicated ion channels as possible targets for therapeutics development. Several factors favor the use of ion channels as therapeutic targets, including aberrantly expressed ion channels is frequently observed in cancer cells; ion channel expression fluctuates with tumor types and tumor stages; antibodies against ion channels can be developed since most of them are extracellular. Increasing evidence suggests that the members of TRP (transient receptor potential) superfamily can function as channels controlling ion transmission across the cell membrane. Currently, the TRP family in vertebrates comprises eight subfamilies [6, 7]. TRP channel dysfunction is associated with a spectrum of disorders [8]. However, the role of TRP channels as an anticancer drug target remains to be explored. Recent collectively hinted potential links between cancer metastasis and the nuclear factor of activated T (NFAT)/ TRPM7/calcineurin signaling in HNSCC. Lines of evidence suggested the oncogenic role of TRPM7 and it as a therapeutic target [9, 10]. We hypothesized that TRPM7 dysfunction is involved in the malignancy development of chemotherapy resistance in HNSCC. This translational study focused on the identification of the functions and regulatory mechanisms of TRPM7 potentially induced through the calcineurin/NFAT pathway, which mediates metastasis and chemotherapeutic resistance in HNSCC. The results should provide a strong rationale for developing therapeutic strategies based on candidate molecules involved in TRPM7 regulation to overcome chemoresistance in cancer cells.

## MATERIALS AND METHODS

### HNSCC tissue samples and cell lines culture

The Affiliated Institutional Review Board (IRB) of Tri-Service General Hospital (TSGH) officially accepted this study (IRB:2-108-05-124), which was conducted in conformance with the Helsinki Declaration for biomedical research principles. Tissue samples were collected from the TSGH tissue archive and retrospectively analyzed after informed consent was acquired from participants. Tissue samples from 96 individuals with both normal and HNSCC tissues were used to generate our tissue array (60 normal tissues and 36 HNSCC tissues). Immunohistochemical (IHC) labeling of tissue arrays was used to assess TRPM7 expression in primary HNSCC. The expression of TRPM7 and associated genes in head and neck cancer was also investigated by using *in silica* data from The Cancer Genome Atlas (TCGA) dataset. We also included other dataset that was reposited in public portal of Gene Expression Omnibus (GEO) databases (accessed in: https://www.ncbi.nlm.nih.gov/geo/) under accession number of GSE26549. Expression Project for Oncology (expO) is intended to integrate gene expression data with longitudinal clinical annotation for creating a uniquely powerful portrait of human malignancies and providing critical insights into diagnostic markers, prognostic indicators, and therapeutic targets. The GSE26549 dataset, which was originally designed to predict the development of oral cancer, was used, and Kaplan–Meier analysis was utilised to evaluate the relationships between gene expression, clinicopathological factors, and survival. Moreover, IHC staining of TRPM7 was performed, and the clinical implications of altered TRPM7 expression was evaluated. Antibody against TRPM7 (1:200, S74-25, Invitrogen, Carlsbad, CA, USA) was adopted as the positive control, and a corresponding concentration of mice immunoglobulin G (IgG) was employed as the negative control, according to standard IHC staining procedure. Two different pathologists looked at TRPM7 expression. TRPM7 protein overexpression was calculated by utilizing the quick score (Q-score) approach (Q = P × I), in which the positive percentage distribution (P) of TRPM7-stained was significant scored from 0% to 100%, while on a four-point scale, intensity (I) of tumor cells were rated and the total score ranged from 0 to 300 [11]. The feed-forward loop of oncogenic activities involved in TRPM7 regulation was also investigated, and the clinical implications were assessed.

### *In vitro* cell culture

The SAS cell lines were stimulated from human squamous cell carcinomas. The FaDu cell line is originally epithelial cells for its lineage and historically retrieved from patient with squamous cell carcinoma originated in hypopharyngeal tumor of a Caucasian male on 1968. For cancer and immuno-oncology studies, this cell line is an excellent transfection host. Several of our cell line was generously provided by Dr. Chun-Shu Lin such as the human OSCC cell lines SAS (derived from poor differentiation of SCC in tongue) and TW2.6 (SCC in the buccal region, which was associated to betel nut consumption and tobacco smoke), and FaDu (SCC of hypopharynx area) while other oral normal human keratinocytes (NHOKs) cell line were collected the American Type Culture Collection (ATCC) (Manassas, VA, USA). OSCC cell lines were preserved in complete medium containing of RPMI-1640 and supplemented with recommendation 10% essential fetal bovine serum (FBS) at 37° C under 5% CO_2_. The complete culture growth medium was replaced regularly for every 48–72 hours. Genetic perturbation *in vitro* model such as overexpression and shRNA-silencing TRPM7 in HNSCC cell lines were used to examine the role of TRPM7; the sulforhodamine B staining (SRB) assay was used to evaluate the cellular viability. We also performed cell migration, invasion, colony formation, and tumorsphere formation assays to determine whether TRPM7 affects biological functions in HNSCC cells.

### Transfection of HNSCC cells with shRNA targeting TRPM7

To observe the role of TRPM7 in regulation of cancer metastasis capacity and chemoresistance, short hairpin RNA (shRNA)-mediated knockdown was employed. The shRNA-mediated lentivirus was acquired from OriGene Corp. (Rockville, MD, USA) and produced according to the manufacturer guidelines. The expression of TRPM7 was intended to knock down by adopting two clones of shRNA: clone #1 (shRNA1, clone ID: RC218043L1V) and clone #2 (shRNA2, clone ID: TL320704). Throughout our accredited BSL-2 laboratory at The Translational Medicine Laboratories, TSGH, shRNA lentivirus construction and transfection were done according to defined standards of practice. The OSCC cells were then further sub-cultured for 5–7 hours at 37° C in a 5% CO_2_ incubator. Either qRT-PCR or Western blotting were used to confirm expression of TRPM7 and validate the efficiency of gene knock-down in the cells. Using the transfected cell batch that resulting in the optimum reduction in expression of respective proteins, we then collected the cells and selected for further experiments.

### Boyden chamber cell migration and invasion

The commercial cell culture inserts from BD Falcon (BD Biosciences, Franklin Lakes, NJ, USA) were utilized in the Boyden specialized compartment for further investigation of the cellular migration capacity and invasion capability. The upper surface of the insert chambers containing FBS-deficient culture medium was seeded with wild-type or shTRPM7 SAS cells, and the bottom chambers were filled with full growth culture medium including 10% FBS. The non-migrated cells in the top section of insert were carefully detached using sterile cotton buds after 24 hours incubation in 5% CO_2_, 37° C incubator. The migrated cells that crossed to the bottom side of compartment were then fixed, co-stained OSCC cell imaging by using 0.1% solution containing of crystal violet (or gentian violet), and thoroughly calculated under the light microscope. The cellular migration study was distinguished from cellular invasion experiment in that there was no precoated-matrigel of the chambers in the migration assay.

### Colony formation assay

A total number of 1 × 10^3^ either wild-type or shRNA-mediated knock down of TRPM7 of the SAS cells were planted in each well of 6-well plates. The cells were then cultured in incubator at condition of 37° C, 5% CO_2_ for about fifteen days. The formed colonies were subsequently rinsed thrice in cold saline, fixed in ice-cold 95% of methanol for about 15 minutes, and co-stained OSCC cell imaging with crystal violet (or gentian violet) for 15 minutes before being final counted. A colony was defined as group of cell that contained of at least 50 cells or more. Six randomly selected areas in each well were tested in triplicate to compute the number of colonies with a diameter of 100 μm.

### Western blotting assay

Prior to blotting, appropriate total protein extraction was performed by washing the OSCC cells in cold 1×PBS, then cellular content was lysed by mixing with RIPA buffer, and cellular protein lysates were obtained using commercially kit for protein extraction (Qiagen, Redwood City, CA, USA). The protein content was the quantified using the Bradford assay (Qiagen, Redwood City, CA, USA) (Cat. No. P0006; Beyotime Biotechnology). A total of 40 μg was prepared by heating the cell lysates and inserted to each gel lane. The protein lysate mixture was then separated on polyvinylidene fluoride (PVDF) membranes by SDS-PAGE electrophoresis, and the incubated with monoclonal antibodies against particular proteins at 4° C condition for overnight. The PVDF membranes were rinsed thrice with PBST containing mixture of 1×PBS and 0.1% Tween® 20 detergent for 10 minutes. The PBST detergent solution was employed for the cleaning step each after incubation with original primary antibodies. The PVDF membranes was then preserved for about two hours at suitable room temperature with antibodies labeled with horseradish peroxidase (HRP) as the secondary antibody before being rinsed three times with PBST for about 10 minutes each time. The enhanced chemiluminescence solution was then employed and BioSpectrum Imaging System were used to detect western blotting bands of the membranes (UVP, Upland, CA, USA).

### Real-time qRT-PCR

Using a TRIzol-based technique extracted total RNA from HNSCC cells, as directed by the manufacturer (Life Technologies, Carlsbad, CA, USA). The total concentration of RNA was estimated by using NanoDrop Nucleic Acid Quantification (Nyxor Biotech, Paris, France). In summary, nearly 1 μg of absolutely RNA were reverse side transcribed by using the RT-PCR kit (Qiagen, Redwood City, CA, USA) containing of the primers, probe mix, and master mix. The PCR was carried out with the SYBR Green PCR kit (Rotor-Gene, Carlsbad, CA, USA) (400; Qiagen, Taipei, Taiwan).

### Tumorsphere formation assay

Our tumorspheres were established by seeding 5×10^2^ wild-type or knockdown of shTRPM7 SAS cells on specialized 6-well plates that retained ultra- low attachment capacity. The cells were cultured under addition of 20 ng/mL bFGF (Corning Inc., Corning, NY, USA), 20 ng/mL of EGF, and B-27 Supplement. The number of tumorspheres (>50 μm) was counted ten days after plating under light microscope; tumorspheres were then harvested and employed in following studies.

### OSCC cell cycle arrest and apoptosis analysis

Flow cytometry method was applied to determine apoptosis induction and alteration of cell cycle. Either wild-type or TRPM7 knock-down of OSCC cells were prepared and incubated with commercially available by using FITC Annexin V Apoptosis or propidium iodide (PI) staining, respectively, as per the supplier’s instructions. The fluorometrically-stained cells were then detected analyzed in BD C6 flow cytometer (Beckman, Fullerton, CA, USA).

### *In vivo* analysis of the effect of TRPM7 in tumor xenograft mouse models

Animal studies were performed in full conformity with TSGH-approved guidelines (approval protocol number IACUC-19-014). The right flanks of NOD/SCID mice (6-week-old female mice, N = 5 per group) supplied from BioLASCO Taiwan Co., Ltd. (Taipei, Taiwan) were injected subcutaneously with a total of 2×10^6^ SAS cells either control wild-type or sh-TRPM7. On a weekly basis, growth of xenografted tumor was observed, and tumor volume was quantified using the conventional caliper method. The equation for determining tumor volume (V) is as follows: tumor volume is equal to (W^2^×L)/2, where the W is primary tumor width and L is the primary tumor length or tumor diameters. The survival curve was represented as Kaplan–Meier plot to evaluate and measure the survival proportion of mice injected with control wild-type and sh-TRPM7 cells. Animals were humanely euthanized through cervical dislocation at the end of the *in vivo* research, and tumor tissues were taken for further analysis.

### Biostatistical analysis

All biostatistical analyses in this study were done finished using GraphPad prism. The Kaplan–Meier plots were generated and used in the survival study. To compare numerical values among two distinct groups, the Student's t test was analyses, while one-way biostatistical analysis of variance (ANOVA) were utilized to compare numerical parameter of several groups (more than 2 groups). All of the statistical tests were carried out in triple repetitions. The mean was used to represent numerical data with standard error of the mean (SEM) was shown to denote deviation level of data. A statistically significance criteria of p value less than 0.05 was used in this study.

### Ethics approval and consent to participate

This study was approved by the Institutional Review Board of the Tri-Service General Hospital (IRB:2-108-05-124) and was conducted according to the recommendations of the Declaration of Helsinki for biomedical research. Animal studies were performed in full conformity with TSGH-approved guidelines (approval protocol number IACUC-19-014).

### Availability of data and materials

The datasets used and analyzed in the current study are publicly accessible as indicated in the manuscript.

## RESULTS

### Expression of TRPM7 and NFATC3 mRNA in the calcineurin/NFAT pathway is upregulated in HNSCC

**Figure 1:**
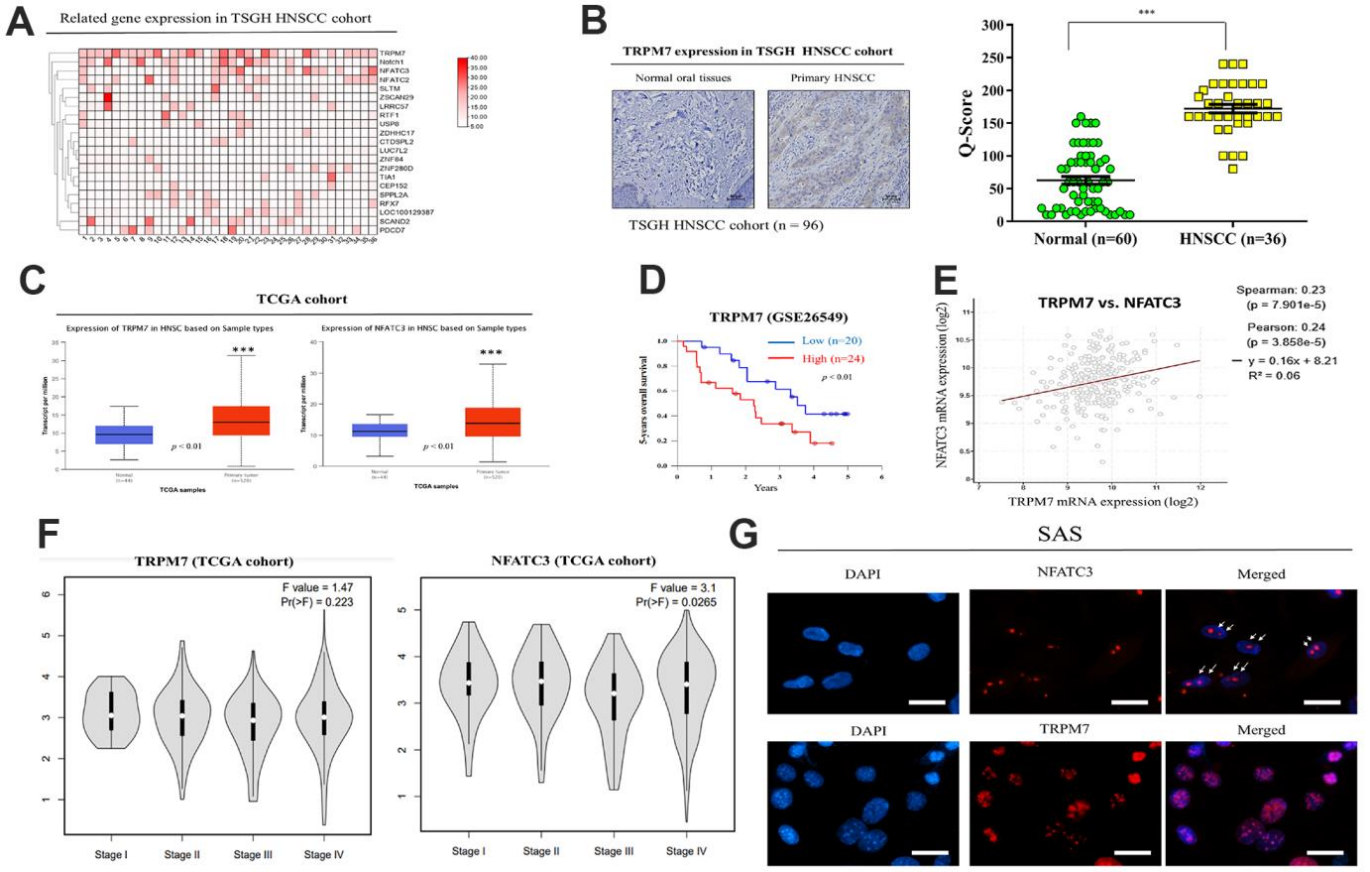
**Expression and prognostic value of TRPM7 mRNA for HNSCC based on the TSGH and TCGA cohort.** (**A**) Heatmap of TRPM7 expression in Tri-Service General Hospital (TSGH) patients with head and neck squamous cell carcinoma. (**B**) Immunostaining showed that TRPM7 expression in the tissues of HNSCC patients was higher than that in normal tissues. (**C**) Box-plot diagrams of TRPM7 and NFATC3 expression in TCGA. (**D**) Using the GSE26549 dataset (n = 44), we demonstrated that high TRPM7 mRNA expression is associated with worse 5-year overall survival compared with low TRPM7 mRNA expression. (**D**) Overall survival of HNSCC patients with a high NFATC2 expression had a poor overall survival rate from the TCGA cohort (**E**) A positive correlation was observed between the expression of the calcineurin/NFAT pathway-related targets NFATC3 and TRPM. (**F**) Expression analysis of TRPM7 and NFATC3 with tumor stage from the TCGA cohort (Image generated from: http://gepia2.cancer-pku.cn/#analysis). (**G**) Immunofluorescence assay to show the co-localization of TRPM7 and calcineurin/NFAT in SAS cells. *p < 0.05, **p < 0.01, ***p < 0.001.

The relationship between TRPM7 expression in human samples and clinicopathological characteristics of TSGH HNSC patients was presented in Table 1. Immunohistochemistry labeling was applied to quantify protein expression using a tissue array comprising of HNSC clinical specimens from 36 individuals. The TRPM7-high and TRPM7-low expression groupings demonstrated substantial differences in tumor differentiation, tumor size, lymphatic node metastatic status, and clinical stage. The expression of TRPM7 in TSGH HNSCC tissue was depicted in Figure 1A. A heatmap of the gene expression patterns collected from 36 HNSCC tissue samples was constructed using the robust microarray analysis expression data for HNSC samples from the TSGH cohort. TRPM7 and NFATC3 had increased levels of expression in HNSC samples than other genes. TRPM7 expression was greater in HNSCC tissues than in normal tissues, according to immunostaining data (Figure 1B). IHC staining of the TRPM7 protein revealed that compared with the weak or no TRPM7 immunostaining in normal oral tissues (n = 60), strong TRPM7 signals were detected in primary HNSCC tissue sections (n = 36; p < 0.01; Mann–Whitney U test). Furthermore, we evaluated TRPM7 and NFATC3 expression in the Gene Expression HNSCC cohort by using the TCGA online open access cancer microarray platform. As shown in Figure 1C, the resulting expression box-plot indicated that TRPM7 and NFATC3 mRNA expression were greater in the HNSCC samples than in the adjacent normal samples (p < 0.01). We observed that high TRPM7 mRNA expression was significantly associated to poorer 5-year overall survival as opposed to low TRPM7 mRNA expression (p < 0.01; Figure 1D) according to GSE26549 dataset (n = 44). We also found a positive correlation between the expression of calcineurin/NFAT pathway-related targets NFATC3 and TRPM7 (Figure 1E). In addition, we try to associate TRPM7 with tumor stage and metastasis to convince high expression was associated with HNSCC invasiveness. Expression analysis from the TCGA cohort showed that TRPM7 expression levels increased with tumor stage (Figure 1F). Finally, Immunofluorescence assay to show the co-localization of TRPM7 and calcineurin/NFAT in SAS cells (Figure 1G). These findings suggested that elevated TRPM7 and NAFTC3 expression may be essential for the development of HNSCC and concomitant poor outcome.

**Table 1:** Correlation between TRPM7 expression and clinicopathological variables of TSGH-HNSCC patients (n=36).

**Clinicopathological variables**	**No.**	**TRPM7**		**x2**	**p-value**
		**High expression**	**Low expression**		
**Age, years**					
60	**20**	12	8	0.051	0.821
60	**16**	9	7		
**Gender**					
Male	**30**	25	5	6.667	0.010
Female	**6**	2	4		
**Differentiation**					
Well/Moderately	**25**	22	3	0.244	0.621
Poor	**11**	9	2		
**Tumor Size (mm)**					
40	**9**	7	2	3.030	0.082
40	**27**	26	1		
**Lymph node metastasis**					
N0	**3**	1	2	3.740	0.053
N1-N2	**33**	27	6		
**Primary Stage**					
I+II	**20**	13	7	4.250893	0.03923
III+IV	**16**	15	1		
**CCRT**					
YES	**25**	23	2	2.823333	0.092903
NO	**11**	7	3		

### Knockdown of TRPM7/NFAT axis expression by shRNA suppresses cell migration and invasion

**Figure 2:**
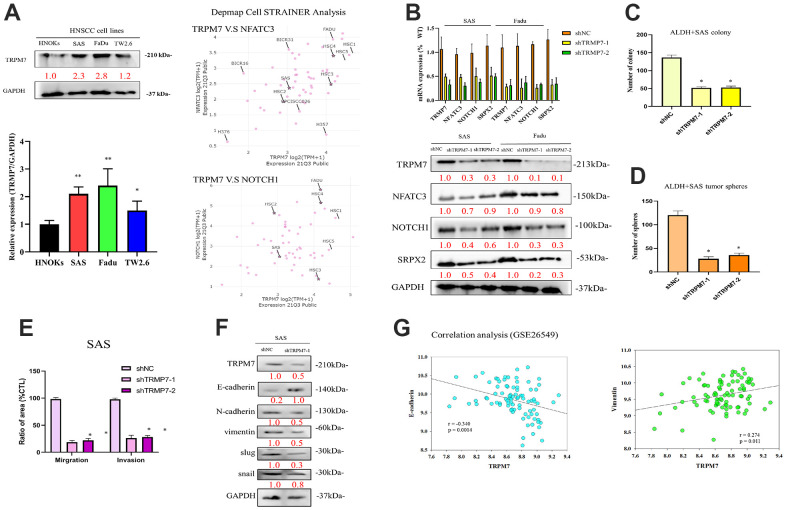
**Expression profiles of TRPM7 in HNSCC-derived cell lines and HNSCC tumor samples as well as knockdown of TRPM7/NFAT expression via shRNA.** (**A**) Immunoblotting analysis of TRPM7 in HNSCC-derived cell lines and NHOKs, quantification of TRPM7 mRNA expression in HNSCC-derived cell lines through qRT-PCR, and target expression screening strategy for head and neck cancer cell lines. (**B**) qRT-PCR and protein analysis of SAS cells with TRPM7 knockdown and control cells in the TRPM7/NFAT axis. Knocking down of TRPM7 downregulated the expression of genes and proteins related to TRPM7 and the calcineurin/NFAT pathway (NFATC3, NOTCH1), which implies that TRPM7 and the calcineurin/NFAT pathway may have a regulatory relationship, as well as representative images of the SAS cell colony and sphere (**C**, **D**). (**E**) Bar graph shows the mean ± standard deviation of the percentage of migrating and invasive SAS cells treated with shNC or shTRPM7 over three independent experiments. (**F**) Western blot analysis results showed target of TRPM7 that may affect invasion and migration pathway of HNSCC by evaluating modulation of several EMT markers. (**G**) Analysis of the relationships of TRPM7 with E-caderin and vimentin. *p < 0.05, **p < 0.01.

In line with the aforementioned findings, our qRT-PCR examination of TRPM7 mRNA expression indicated that, as compared to NHOKs, TRPM7 mRNA was overexpressed in SAS (5.62-fold, p < 0.01), FaDu (7.75-fold, p < 0.01), and TW2.6 (7.84-fold, p < 0.01) cells. Furthermore, in agreement with the results of the qRT-PCR, TRPM7 protein overexpression was significantly increased in FaDu (5.01-fold, p 0.01), SAS (7.99-fold, p 0.01), and TW2.6 (7.84-fold, p 0.01) cells than in NHOKs, as demonstrated by representative Western blot study. After analyzing the overexpression of TRPM7, NFATC3, and Notch1 within different head and neck cancer cell lines, we screened cell lines suitable for subsequent experiments. We use open source tools online to analyze target expression in cells (https://depmap.org/portal/). The results showed that SAS and FaDu cells exhibited TRPM7/NFAT axis expression. These two cell lines were used in subsequent experiments (Figure 2A). To understand the effect of altered TRPM7 expression in HNSCC, we knocked down TRPM7 expression in SAS and FaDu cells by using shRNA (Figure 2B). The results showed that knockdown of TRPM7 expression downregulated the expression of genes and proteins related to TRPM7 and the calcineurin/NFAT pathway (NFATC3, NOTCH1), which implies a regulatory relationship between TRPM7 and the calcineurin/NFAT pathway (Figure 2B). shTRPM7-1 and shTRPM7-2 significantly reduced the percentage of migrating (75%–81% reduction, p < 0.01) and invasive (72%–83% reduction, p < 0.01) SAS cells compared with their shNC counterparts (Figure 2C–2E). Western blot analysis results showed target of TRPM7 that may affect invasion and migration pathway of HNSCC by evaluating modulation of several EMT markers. TRPM7 depletion modulates the de-activation of EMT markers throughout cancer cells (Figure 2F). TRPM7 was negatively correlated with the epithelial marker E-cadherin (r = 0.340, p = 0.0014), whereas it had a positive moderate correlation with the mesenchymal metastatic marker, namely vimentin (r = 0.274, p = 0.011) in our analysis of GSE26549 dataset that determined the relationships between TRPM7 and classical epithelial-to-mesenchymal transition markers (Vimentin and E-cadherin) (Figure 2G). According to these results, TRPM7 expression was linked to highly aggressive and metastatic phenotypes of HNSCC.

### Downregulation of TRPM7 expression inhibits cancer stemness and colony formation in HNSCC

**Figure 3:**
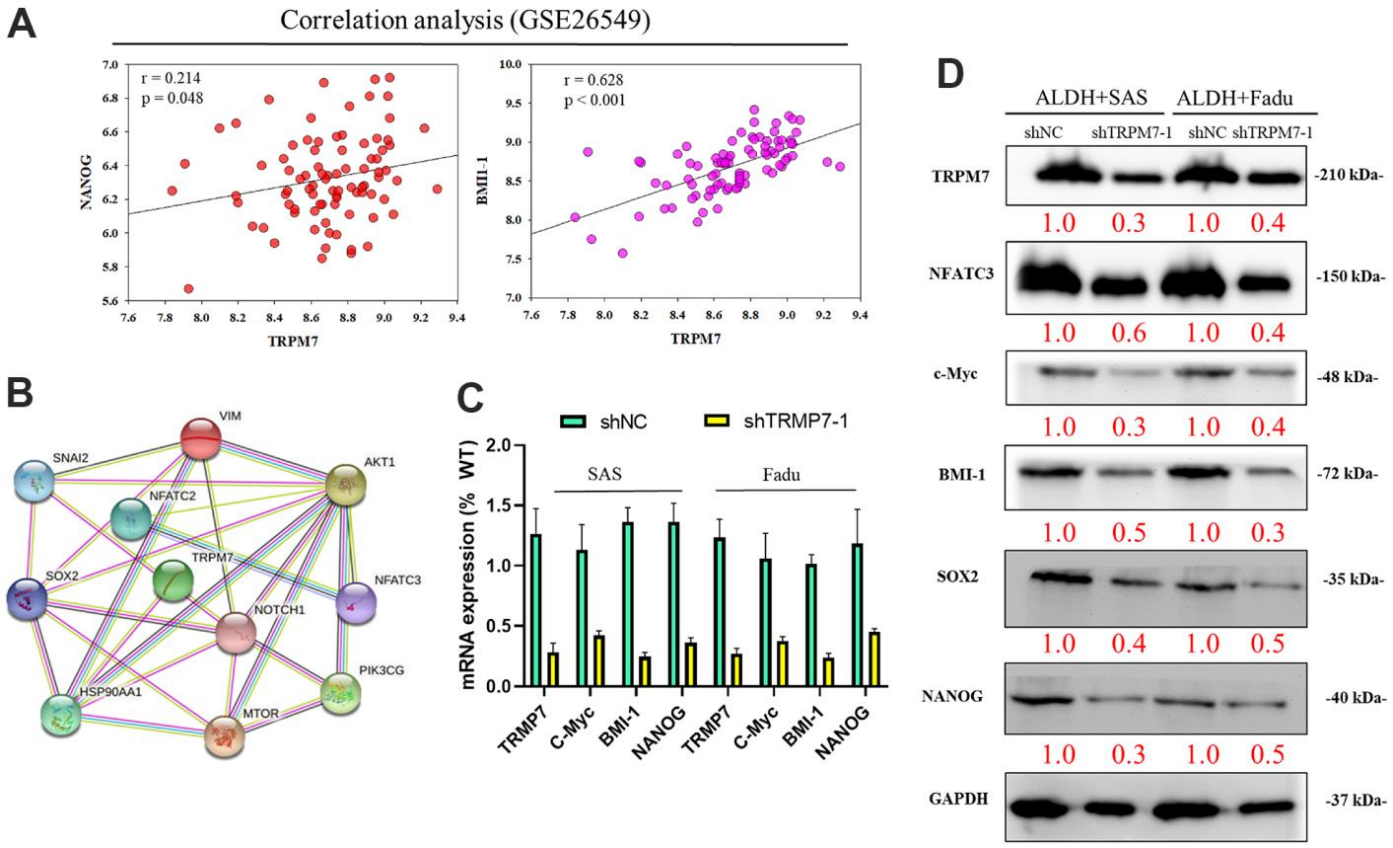
**Downregulation of TRPM7 expression inhibits cancer stemness cell proliferation-associated protein in HNSCC.** (**A**) Analysis of the relationship of TRPM7 with NANOG and BMI-1 (**B**) The possible regulation network of TRPM7. (**C**) Reduced mRNA expression of TRPM7, c-Myc, BMI-1, and Nanog proteins and TRPM7 knockdown in SAS and FaDu cells based on qPCR analysis. (**D**) Western blots of tumorspheres derived from HNSCC cells showing the expression profiles of TRPM7, NFATC3, c-Myc, BMI-1, Sox2, and Nanog. *p < 0.05, **p < 0.01.

Using the GSE26549 oral cancer cohort (n = 49), we also demonstrated mild or strong positive correlations between TRPM7 and NANOG (r = 0.214, p = 0.048) or BMI-1 (r = 0.628, p < 0.001) (Figure 3A). Figure 3B shows the possible regulatory network of TRPM7. Furthermore, silencing TRPM7 (~5.0-fold, p < 0.05) was significantly downregulated the overexpression of c-Myc (2.5-fold, p < 0.05), BMI-1 (3.0-fold, p < 0.05), and NANOG (3.3-fold, p < 0.05) in SAS and FaDu cells (Figure 3C). In addition, Western blot analysis revealed that TRPM7 downregulation in SAS and FaDu tumorsphere-derived cells induced significant downregulation of the TRPM7/NFAT axis; Snail, c-Myc, and BMI-1 proteins, and the stemness markers Sox2 and Nanog (Figure 3D). These results indicate, at least partially, that TRPM7/NFAT axis has a vital regulatory role in HNSCC stemness and progression. To better demonstrate the role of TRPM7 on NFAT pathway, in transient TRPM7-knocked down cancer cell model. We treat the cells with NFAT activators positive control (as PMA and calcium ionophore) as well. This analysis demonstrated that combined PMA and ionomycin treatment resulted in blocked NAFTC3–dependent activation in SAS cells (Supplementary Figure 1).

### High TRPM7 expression is associated with cisplatin resistance, but shRNA-mediated TRPM7 silencing synergistically enhances cisplatin anticancer cytotoxicity

**Figure 4:**
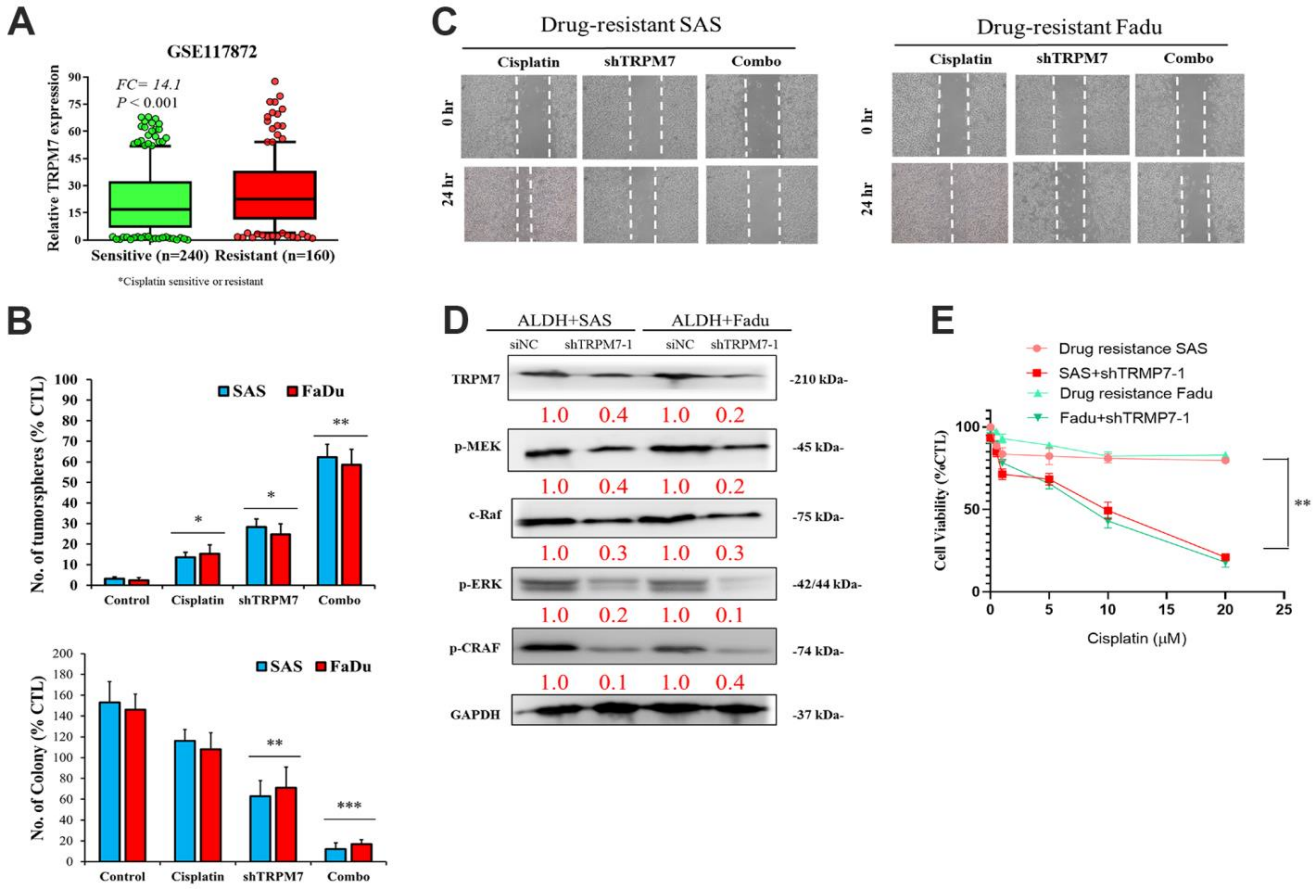
**Combination of anti-TRPM7 shRNA and cisplatin enhances cytotoxicity in ALDH+ HNSCC cells.** (**A**) Box-plot diagrams of the relationship of TRPM7 expression with cisplatin sensitivity/resistance according to the GEO dataset GSE117872. (**B**) Significant inhibitory effect of the combination of TRPM7 shRNA and cisplatin on tumorspheres. (**C**) Wound healing analysis by using cells transfected with TRPM7 shRNA and cisplatin in SAS and FaDu cells. (**D**) Association of TRPM7 with possible regulatory pathways in Western blot assays. (**E**) Apoptosis analysis of SAS and FaDu cells with or without TRPM7 shRNA.*p < 0.05, **p < 0.01. Scale bar: 100 μm.

Analysis of the GSE117872 dataset (n = 400) revealed that compared with the cisplatin-sensitive group (n = 240), higher TRPM7 expression was found in the cisplatin-resistant HNSCC group (n = 160) (1.32-fold, p < 0.001) (Figure 4A). The tumorsphere formation assay showed that compared with untreated wild-type SAS cells, treatment with 5–20 μM cisplatin alone caused a nonsignificant mild reduction (1.18-fold) in the number of tumorspheres formed, and treatment with shTRPM7 alone (2.24-fold, p < 0.01) or in combination with 10 μM cisplatin (10.83-fold, p < 0.001) significantly suppressed the formation of SAS tumorspheres. A similar inhibitory trend was also found for the ability of SAS and FaDu cells to form colonies upon treatment with 10 μM cisplatin or shTRPM7 (Figure 4B). Collective migration in the wound healing assay showed that the combination therapy could reduce the migration ability of drug-resistant cell lines (Figure 4C). In addition, Western blot analysis showed that TRPM7 downregulation in SAS and FaDu tumorsphere-derived cells induced significant downregulation of the TRPM7/NFAT axis; Snail, c-Myc, and BMI-1 proteins; and the stemness markers Sox2 and Nanog (Figure 3D). Western blot analysis also revealed that TRPM7 downregulation in SAS and FaDu tumorsphere-derived cells induced significant downregulation of MEK, C-RAF, ERK, and CRAF activities (Figure 4D). Furthermore, the results of our cytotoxicity/tumorsphere viability assay showed that relative to untreated wild-type control, 24-h treatment with 1 μM cisplatin alone caused 13% (p < 0.05) and 15% (p < 0.05) increases in the percentage of apoptotic SAS and FaDu cells, respectively, and treatment with shTRPM7 alone resulted in 28% (p < 0.05) and 25% (p < 0.05) increases in the percentage of apoptotic SAS and FaDu cells; moreover, combining 1 μM cisplatin with shTRPM7 significantly enhanced the apoptosis of SAS (62%, p < 0.01) and FaDu (59%, p < 0.01) cells (Figure 4E). These data indicate that targeting TRPM7 synergistically enhances the anti-HNSCC effect of cisplatin.

### TRPM7 silencing significantly suppresses metastasis in the SAS-derived tumor xenograft model *in vivo*

**Figure 5:**
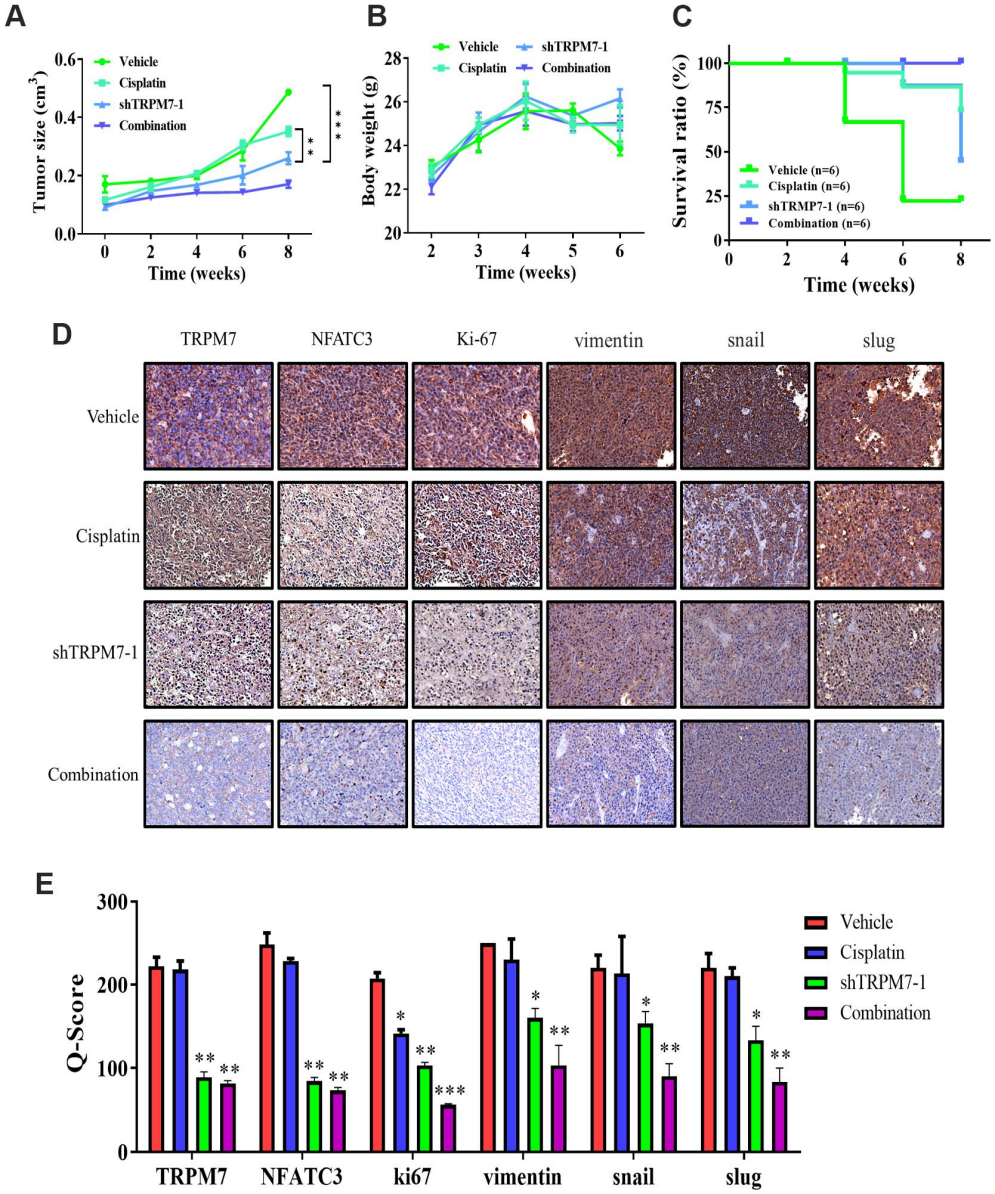
**TRPM7 silencing significantly suppresses metastasis in SAS-derived tumor xenograft model *in vivo*.** (**A**) The tumor curve over time shows that cisplatin and shTRPM7 in combination suppressed tumor growth. (**B**) The average body weight over time curve demonstrated no apparent systematic toxicity in the mice receiving combined treatment. (**C**) The Kaplan–Meier survival curve shows that the mice receiving combined treatment had the highest survival ratio compared to the other groups. (**D**) Immunostaining analysis of tumor sections showed that the combined treatment suppressed NAFTC3 and ki67 expression the most, compared with other groups. **P < 0.01; ***P < 0.001. (**E**) Bar graph showing the comparison of Q-score for tissue expression in tumor section resulted significant inhibition of NFATC3, ki67, and EMT markers vimentin, snail, and slug. *p < 0.05, **P < 0.01, and ***P < 0.001.

SAS-derived xenograft mouse models were created by orthotopic inoculation of wild-type or TRPM7-knock down SAS cells forward into right flank of NOD-SCID female mice for *in vivo* validation of the findings gained from *in vitro* study. The tumors that developed in mice implanted with TRPM7-silenced cells were markedly smaller at indicated time points compared with the control mice, with a 1.6-fold difference in tumor size by week 8 (p < 0.01) (Figure 5A). Furthermore, the mice with TRPM7-knockdown tumor cells had a considerably higher survival rate, yet there was no significant impact on the mice's bodyweight during week 6 (Figure 5B, 5C). Using tumor samples derived from the tumor xenograft mouse models, we demonstrated that the expression of TRPM7, NFATC3, and ki67 proteins was significantly suppressed in the TRPM7-silenced and combined treatment mice compared with that in the control mice. The Q-score of tissue staining was also calculated. These findings indicated that TRPM7 plays a crucial role in malignant progression of HNSCC as well as in the modulation of markers (Figure 5D, 5E).

## DISCUSSION

The incidence of HNSCC has continued to increase rapidly over the past decade in Taiwan, and the prognosis has remained poor, mostly due to treatment failure and distant metastases [12]. Surgical resection remains the main treatment modality. Concurrent chemoradiotherapy is used for eradicating local HNSCC if patients are not eligible for surgery [13]. Generally, treatment outcomes have remained unchanged in the last three decades [12–14] while limited new therapeutic options are available [14]. Thus, HNSCC remains an unmet clinical need in Taiwan.

The present study explored the role of TRPM7 in HNSCC progression. The TRP melatonin-like (TRPM) family consists 8 members (TRPM1–TRPM8) each member with different physiological functions and implicated in different disorders [15, 16]. TRPM7 plays a critical role in cell and developmental biology, as well as in human diseases [17, 18]. TRPM7 has been under investigation and its functions have been uncovered in different cell models [19]. TRPM7 channel kinase functions as sensor for physical and chemical stress [20]. Studies have revealed the oncogenic role and therapeutic targeting of TRPM7 in different cancer types [21–24]. However, up-to-date the precise role of TRPM7 in HNSCC remains unclear. Hypothesizing that the aberrant expression of TRPM7 is involved in chemoresistance development in HNSCC cells, we identified regulatory mechanisms of TRPM7 in HNSCC. Our analysis of several GEO datasets (GSE12452, GSE2109, and GSE9844) showed that *TRPM7* mRNA expression was upregulated in HNSCC, including NPC, tongue SCC, and hypopharyngeal SCC; high *TRPM7* expression was associated with poor prognosis in HNSCC patients (Figures 1, 2). These findings are consistent with those of a recent report that TRPM7 overexpression was significantly higher in renal cell carcinoma clinical samples and RCC cancer cell lines, and this was positively correlated with tumor stages and worse overall survival and progression-free survival among RCC patients [25].

Squamous cell carcinoma (SCC), which includes skin cancer, oral and esophageal cancer, squamous (bronchial) lung cancer, cervical cancer, and cancers of various areas of the genitourinary system, has a very high prevalence in the population. These tumors are notable for their heterogeneity, comprising of self-renewing stem cell groups and cells at different stages of development. The dynamic balance between the stem cell population and its daughter cells may influence the aggressiveness of these tumors and their capacity to restore after therapy. The deregulation of calcineurin/NFAT signaling has been linked to cancer in several studies. This pathway is in charge of tumor angiogenesis. Increased calcineurin activity leads to endothelial cell death and tumor suppression in mice with calcineurin deficiency. As a result of calcineurin activation, one of the dephosphorylated proteins is NFAT. The phosphorylation of NFATs by various kinases such as GSK3, JNK1, and DYRK1A prevents nuclear localization of these factors. Cancer cell senescence appears as a fail-safe strategy for hindering tumorigenesis and the capacity of cancer stem cells. Calcineurin/NFAT inhibition was demonstrated to suppress cancer cell senescence and downregulate p53.

In this study, we provided preclinical evidence that silenced TRPM7 expression significantly suppressed the metastatic trait of HNSCC cells, where the migration and invasion abilities of HNSCC cells were reduced and was associated with a decreased vimentin/E-cadherin ratio (Figure 2). Vimentin is a master regulator of cell migration and EMT [26]. EMT is an essential event during tumor progression. Furthermore, a recent study found that suppressing TRPM7 prevented hypoxia-induced malignant migration and invasion of androgen-independent prostate cancer cells via increasing RACK1-mediated HIF-1alpha degradation [27]. Another study demonstrated that cell invasion is markedly suppressed by silencing TRPM7, which was accompanied by the reduced secretion of EMT mediators, such as MMP2, uPAR, and HSP90 [28]. These findings strongly support TRPMs’ participation in cellular migration, invasion, and resistance to chemotherapy [21].

In the context of malignancies, TRPM7 is a Ca^2+^ and Mg^2+^ permeable ion channel which is associated with the growth and progression in breast, gastric, pancreatic, and prostate cancers [29]. Elevated TRPM7 expression in human breast and pancreatic cancer tissues has been implicated in advanced tumor grade, proliferation, and poor survival rates [29, 30]. Various studies support our speculation that TRPM7 plays a crucial role in the metastasis of breast cancer, and it is an independent marker of poor prognosis [30]. In addition, high TRPM7 expression in patients with neuroblastoma has been linked to metastatic propensity [31] Furthermore, TRPM7 expression has been linked to EMT [18]. We showed that enhanced expression of vimentin and phosphorylated/activated STAT3 might be associated with enhanced intracellular Ca^2+^flux induced by stemness-related hypoxia [32]. A recent study showed that TRPM7 was necessary for breast cancer metastasis in a mouse model [33]. This is in line with our findings where TRPM7 was linked to HNSCC EMT and metastasis; TRPM7 was required to maintain HNSCC stem cell-like trait and involved in chemoresistance (Figure 3). Another supporting study showed that TRPM7 overexpression resulted in the increased expression of cancer stemness markers such as SOX2, KLF4, and CD133 in lung cancer, while TRPM7-silencing led to the reduction of stemness and EMT traits [34, 35].

Cisplatin, a platinum-containing anticancer drug, has been used as the first-line chemotherapeutic agent for treating head and neck cancer [36, 37]. Unfortunately, many patients develop cisplatin resistance, leading to cancer recurrence. With increased understanding of the cisplatin resistance mechanism, combination therapy has become a common alternative treatment strategy [36, 37]. We provide preclinical evidence that TRPM7 overexpression is associated with cisplatin resistance, and that shRNA-mediated TRPM7 silencing synergistically enhances cisplatin cytotoxicity of cancer cells (Figure 4). These data indicate that targeting TRPM7 sensitizes HNSCC to cisplatin; altered TRPM7 expression, independent of or within the context of calcium signaling pathway induction, is involved in HNSCC cell proliferation, invasion, migration, evasion of cell death signaling, remodeling of the extracellular matrix, EMT, and chemoresistance. Consistent with the existing data, growing evidence indicates that calcium signaling dysregulation by TRP channels such as TRPM7 drives cancer growth, metastasis, and resistance to chemotherapeutics, including cisplatin [38]. Thus, our finding that targeting TRPM7 enhances HNSCC cells’ sensitivity to cisplatin, represents an additional armament to overcoming drug resistance [38]. When compared to their control counterparts, TRPM7-silenced tumor samples had significantly lower expression of NFATC2, Notch1, and P-MEK proteins (Figure 5). A functional calcineurin/NFAT signaling is essential for preventing cutaneous squamous cell carcinoma development. Orai1, a key calcium channel, has been implicated in human cancer. The effect of Orai1 on CSCs trait was reversed when NFATc3 was silenced in Orai1-overexpressing oral epithelial cells [39]. Furthermore, antagonists of NFAT signaling suppressed the phenotype cancer stem-like cells, indicating that calcineurin/NFAT signaling is important for OSCC CSCs maintenance. We hypothesized that the TRPM7-mediated calcineurin/NFAT pathway is important for HNSCC carcinogenesis throughout this investigation. In SAS-injected tumor xenograft mice models, TRPM7 silencing resulted in considerably reduced tumor burden, decreased metastasis, and higher survival rates. TRPM7 knockdown reduced the expression of all metastasis-associated markers, suggesting that TRPM7 might be a new therapeutic target for attenuating HNSCC metastasis and chemotherapeutic resistance.

## CONCLUSIONS

**Figure 6:**
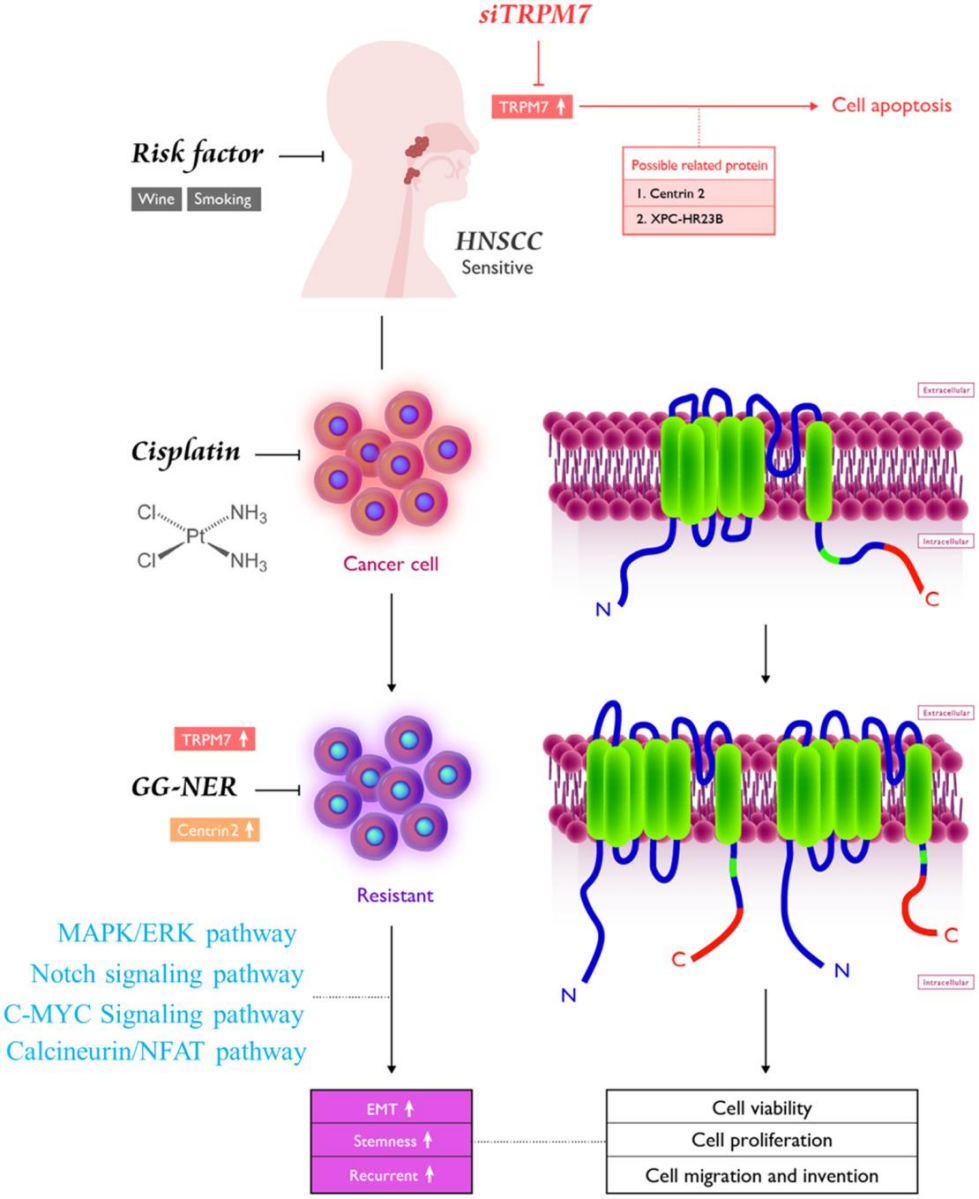
Pictorial abstract showing the essential role of TRPM7 in the metastasis and chemoresistance of HNSCC cells; silencing TRPM7 suppressed several oncogenic signaling axes, leading to reduced migration, invasion, colony formation, and spheroid formation.

As shown in the pictorial abstract in Figure 6, TRPM7 silencing suppressed several oncogenic signaling axes, leading to reduced migration, invasion, colony formation, and spheroid formation. Because of the effect of TRM7-regulated divalent cations, the scope of influence may be multilevel [18, 22, 29, 34]. The present study demonstrated that targeting TRPM7 may be an effective treatment strategy for cisplatin-resistant HNSCC. This is of therapeutic relevance considering that the last decade has been characterized by some reports on the development of TRPM7 inhibitors [40, 41]. Finally, the present study provides some rationale for developing effective therapeutic strategies based on targeting TRPM7, its modulators or downstream mediators, to overcome chemoresistance in HNSCC.

## Supplementary Material

Supplementary Figures
