# Supplementary material for: TRPM7 via calcineurin/NFAT pathway mediates metastasis and chemotherapeutic resistance in head and neck squamous cell carcinoma
Source: Aging (Albany NY). 2022 Jun 29;14(12):5250–70. doi: 10.18632/aging.204154 (PMC9271301; doi:10.18632/aging.204154)
Supplement: Supplementary Figures [file aging-14-204154-s001.pdf]

SUPPLEMENTARY FIGURES

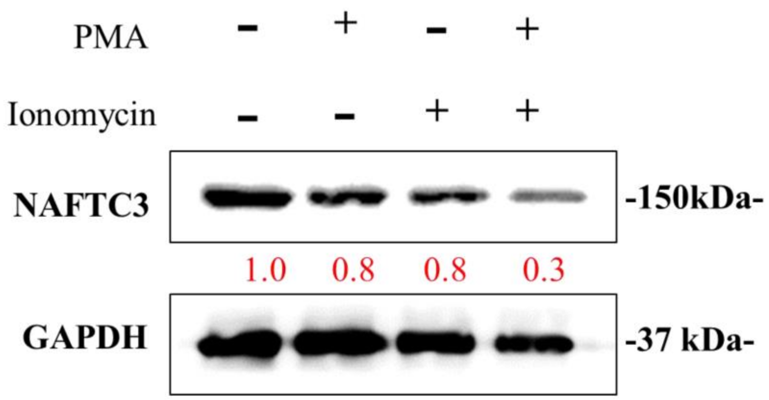

Supplementary Figure 1. Immunoblotting analyses of NFATc3 protein expression levels in SAS cells. Cells stimulated with PMA (20 ng/ml), ionomycin (1 mg/ml) for 60 min.

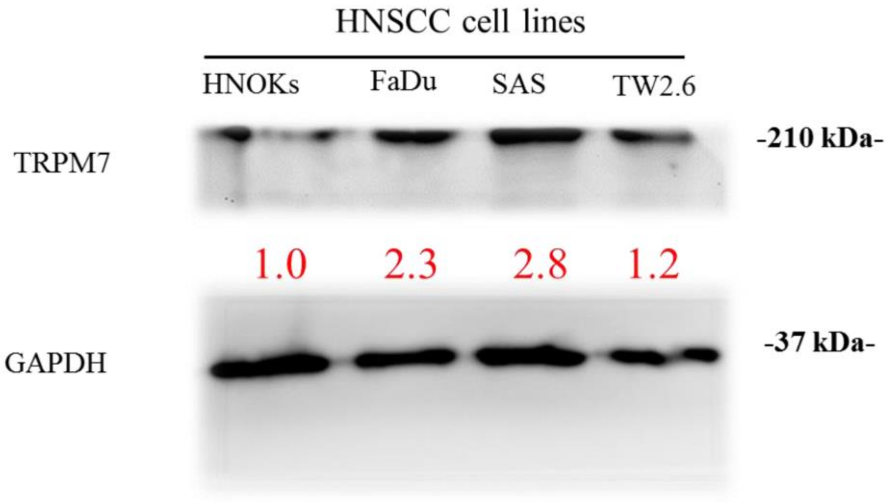

Supplementary Figure 2. Full-size blots of Figure 2A.

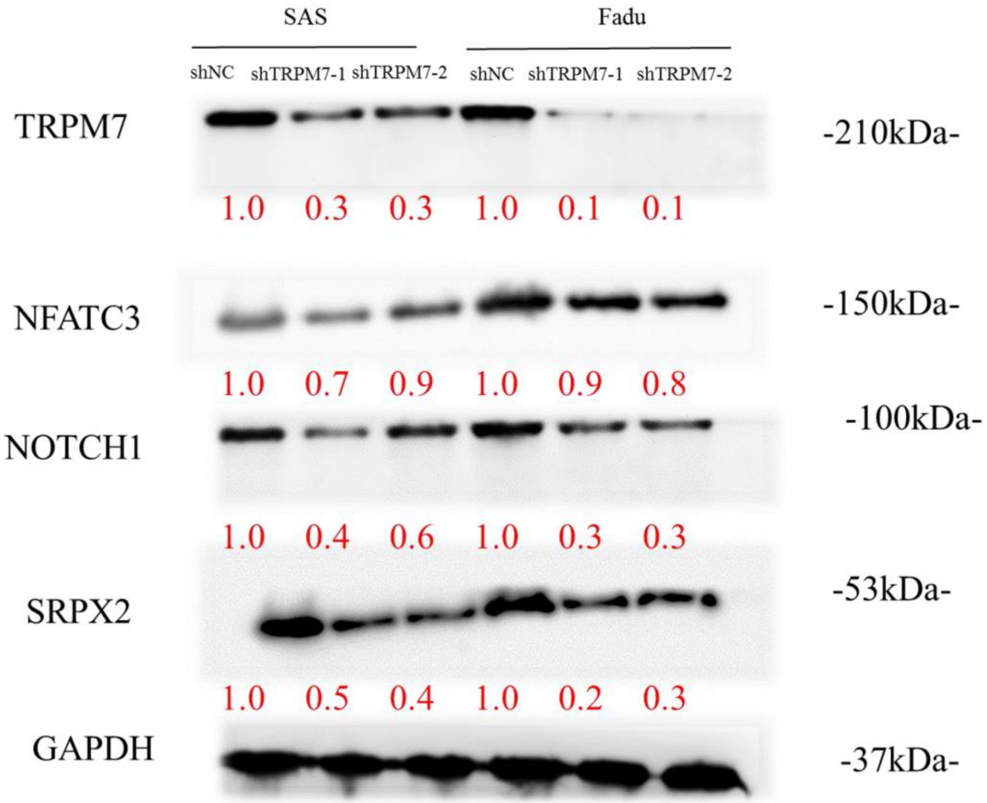

Supplementary Figure 3. Full-size blots of Figure 2B.

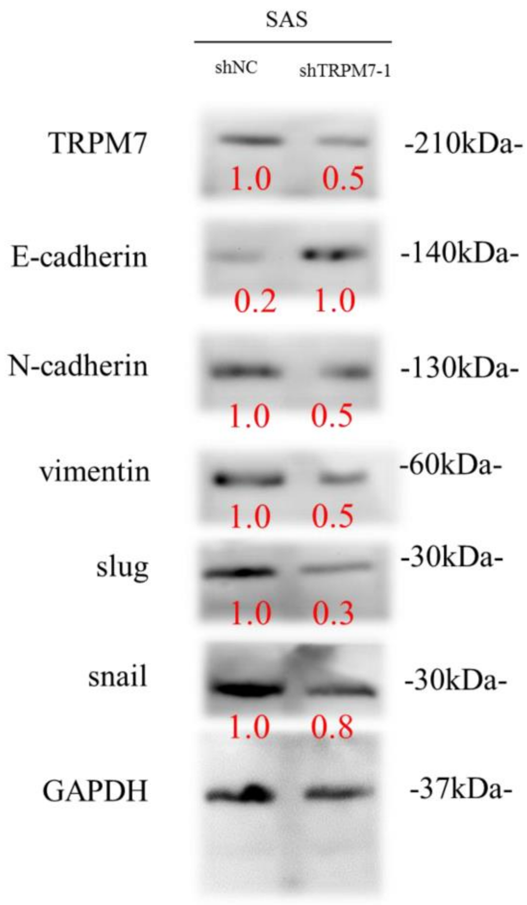

Supplementary Figure 4. Full-size blots of Figure 2F.

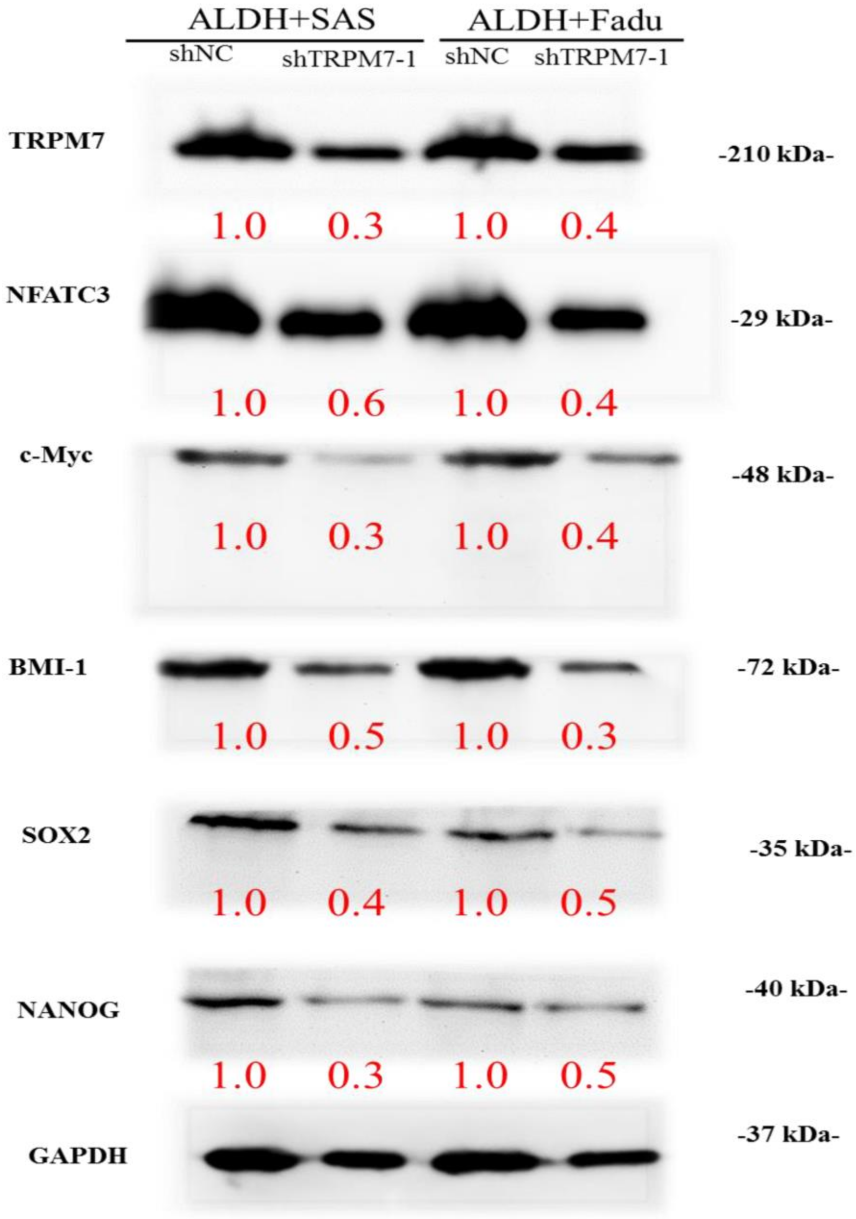

Supplementary Figure 5. Full-size blots of Figure 3D.

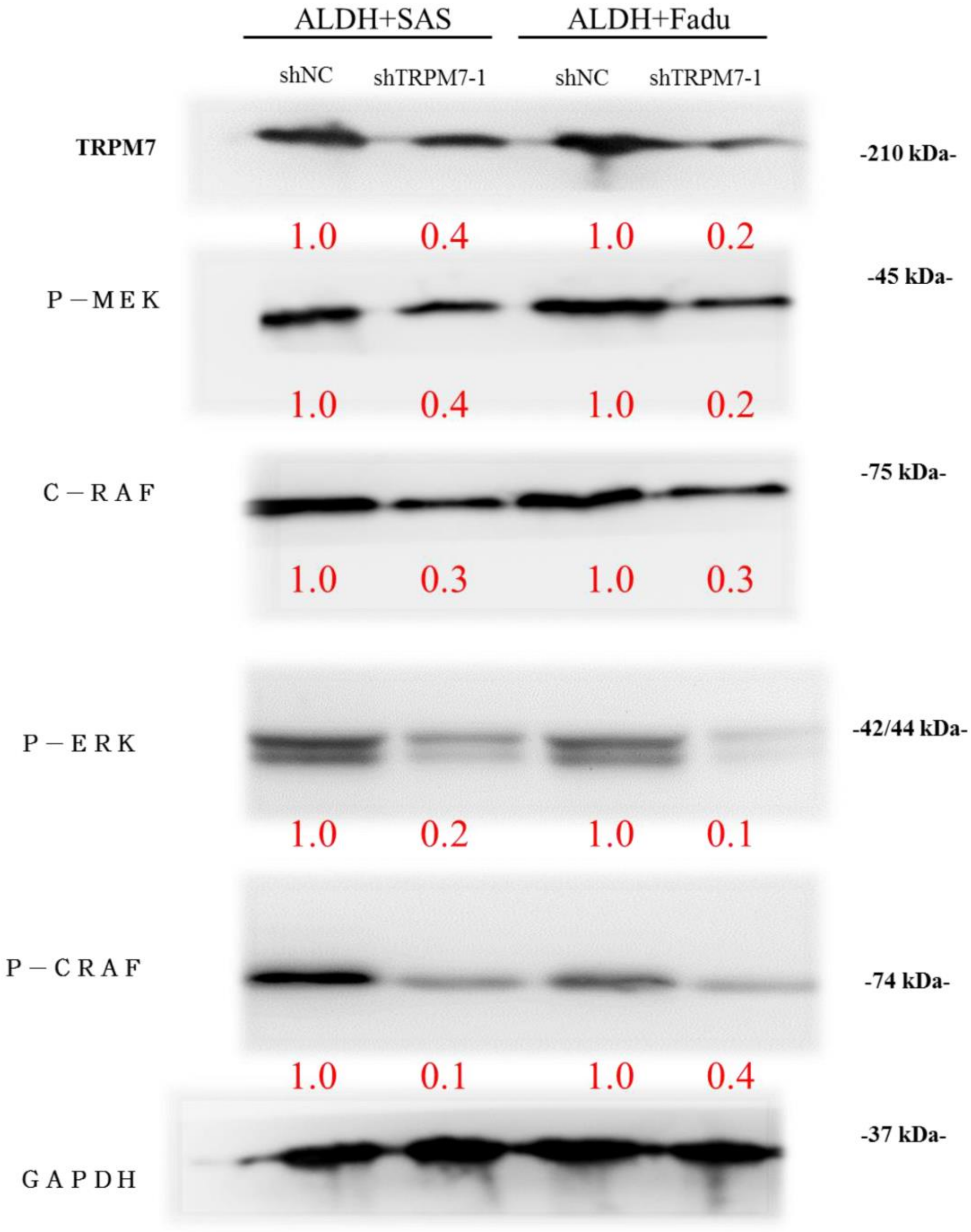

Supplementary Figure 6. Full-size blots of Figure 4D.
